# Evolutionary Conservation of the Orchid MYB Transcription Factors DIV, RAD, and DRIF

**DOI:** 10.3389/fpls.2019.01359

**Published:** 2019-11-01

**Authors:** Maria Carmen Valoroso, Rómulo Sobral, Giuseppe Saccone, Marco Salvemini, Maria Manuela Ribeiro Costa, Serena Aceto

**Affiliations:** ^1^Department of Biology, University of Naples Federico II, Naples, Italy; ^2^BioSystems & Integrative Sciences Institute (BioISI), Plant Functional Biology Centre, University of Minho, Campus de Gualtar, Braga, Portugal

**Keywords:** DIVARICATA, RADIALIS, DRIF, MYB, Orchidaceae

## Abstract

The MYB transcription factors DIVARICATA (DIV), DIV-and-RAD-Interacting-Factor (DRIF), and the small interfering peptide RADIALIS (RAD) can interact, forming a regulatory module that controls different plant developmental processes. In the snapdragon *Antirrhinum majus*, this module, together with the TCP transcription factor CYCLOIDEA (CYC), is responsible for the establishment of floral dorsoventral asymmetry. The spatial gene expression pattern of the *OitDIV*, *OitDRIF*, and *OitRAD* homologs of *Orchis italica*, an orchid with zygomorphic flowers, has suggested a possible conserved role of these genes in bilateral symmetry of the orchid flower. Here, we have identified four *DRIF* genes of orchids and have reconstructed their genomic organization and evolution. In addition, we found snapdragon transcriptional *cis*-regulatory elements of *DIV* and *RAD* loci generally conserved within the corresponding orchid orthologues. We have tested the biochemical interactions among OitDIV, OitDRIF1, and OitRAD of *O. italica*, showing that OitDRIF1 can interact both with OitDIV and OitRAD, whereas OitDIV and OitRAD do not directly interact, as in *A. majus*. The analysis of the quantitative expression profile of these MYB genes revealed that in zygomorphic orchid flowers, the *DIV*, *DRIF1*, and *RAD* transcripts are present at higher levels in the lip than in lateral inner tepals, whereas in peloric orchid flowers they show similar expression levels. These results indicate that MYB transcription factors could have a role in shaping zygomorphy of the orchid flower, potentially enriching the underlying orchid developmental code.

## Introduction

The MYB proteins DIVARICATA (DIV), RADIALIS (RAD), and DIV-and-RAD-Interacting-Factor (DRIF) are part of a regulatory module involved in distinct developmental processes of plants (Machemer et al., 2011; Raimundo et al., 2013). *DIV* and *DRIF* belong to ancient gene families that emerged in the green algae lineage, whereas the *RAD* genes are more recent as their origin can be dated back to gymnosperms (Raimundo et al., 2018). Canonical DIV transcription factors have two MYB domains (MYBI and II) (Galego and Almeida, 2002), in contrast to RAD and DRIF, both containing a single MYB domain (Corley et al., 2005; Raimundo et al., 2013). In addition to the N-terminal MYB domain, DRIF proteins share the conserved DUF3755 domain at the C-terminus, found only in this protein family and whose ability to bind WUSCHEL-RELATED HOMEOBOX (WOX) and KNOTTED1-LIKE HOMEOBOX (KNOX) proteins has been recently described in *Populus trichocarpa* (Petzold et al., 2018). During evolution, the MYB domain has undergone successive rearrangements resulting in the acquisition of specific interaction abilities: the MYB domain of DRIF can interact with the MYBI domain of DIV or with the MYB domain of RAD (Machemer et al., 2011; Raimundo et al., 2013; Raimundo et al., 2018). In such interaction module, the small RAD proteins (less than 100 amino acids in size) have an antagonistic effect on the formation of the DIV/DRIF complex and thus have been classified as small-interfering peptides (siPEP) or microproteins (Seo et al., 2011; Staudt and Wenkel, 2011; Eguen et al., 2015).

**Figure 1:**
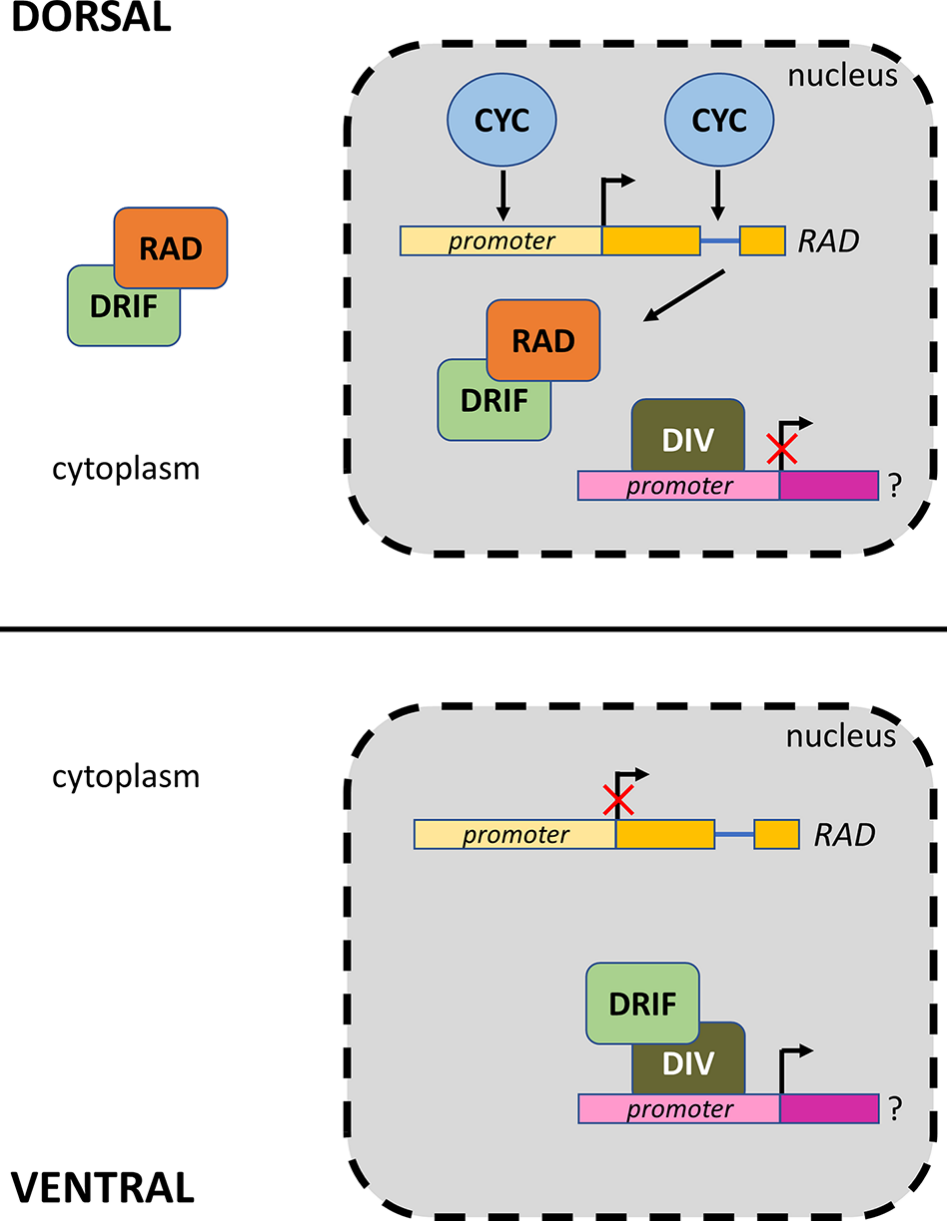
Model of interaction of CYC, DIV, RAD, and DRIF proteins in *Antirrhinum majus* (modified from Raimundo et al., 2013). The DIV and DRIF proteins are expressed both in the dorsal and ventral parts of the flower. In the dorsal domain, the CYC protein interacts with the promoter and intron of the *RAD* gene, activating its expression. The RAD protein binds to DRIF and prevents the formation of the DIV/DRIF complex, thus inhibiting ventralization. In the ventral domain, the absence of RAD permits the formation of the DIV/DRIF complex that controls downstream genes involved in the ventralization.

The function of the DIV, DRIF, and RAD proteins has been described in different plant species, where they control distinct developmental processes. For example, RAD-like proteins regulate photomorphogenesis and floral transition of *Arabidopsis thaliana* (Hamaguchi et al., 2008; Li et al., 2015), DIV-like proteins are involved in sugar and hormone regulation of *Oryza sativa* (Lu et al., 2002), and the protein complexes DIV/DRIF and RAD/DRIF control cell expansion of the fruit pericarp of *Solanum lycopersicum* (Machemer et al., 2011). However, the majority of studies regarding the *DIV*, *DRIF*, and *RAD* genes focused on their role in the establishment of flower zygomorphy, an evolutionary novelty that emerged several times in flowering plants from the ancestral condition of radial symmetry (Citerne et al., 2010; Endress, 2012). The first comprehensive analysis of the molecular pathway underlying floral symmetry was conducted in the snapdragon *Antirrhinum majus* (**Figure 1**), showing that mutations of the genes *CYCLOIDEA* (*CYC*), *DIV*, and *RAD* have an effect on symmetry of the flower. The TCP transcription factor *CYC* is expressed in the dorsal part of the flower and activates the expression of *RAD* (Luo et al., 1996; Cubas et al., 1999; Luo et al., 1999; Corley et al., 2005; Costa et al., 2005) through the interaction with 5′-GGNCCC-3′ binding sites in the *RAD* promoter and intron (Costa et al., 2005). The *DIV* and *DRIF* genes are expressed both in the dorsal and ventral domains of the flower of *A. majus* (Almeida et al., 1997; Galego and Almeida, 2002; Raimundo et al., 2013). In the ventral domain, the protein complex DIV/DRIF controls downstream genes involved in the ventralization of the flower. In the dorsal domain, the siPEP RAD binds to DRIF and prevents its interaction with DIV, thus inhibiting ventralization (Raimundo et al., 2013). In addition to its ability to activate ventralization genes, the DIV/DRIF protein dimers can bind the sequence 5′-GATAA-3′ (Raimundo et al., 2013) within the *DIV* promoter, possibly autoregulating its transcriptional activity (Sengupta and Hileman, 2018).

The role of the *CYC*, *DIV*, and *RAD* genes in controlling flower bilateral symmetry outside *A. majus* has been reported in other Lamiales (Zhou et al., 2008; Preston et al., 2009; Reardon et al., 2009; Preston et al., 2011; Reardon et al., 2014; Su et al., 2017) and in Dipsacales (Howarth and Donoghue, 2009; Boyden et al., 2012), whereas only limited knowledge about these genes is available in monocots and basal angiosperms. However, a recent study suggested the recruitment of this molecular network for the establishment of floral zygomorphy before the diversification between monocots and dicots (Madrigal et al., 2019).

**Figure 2:**
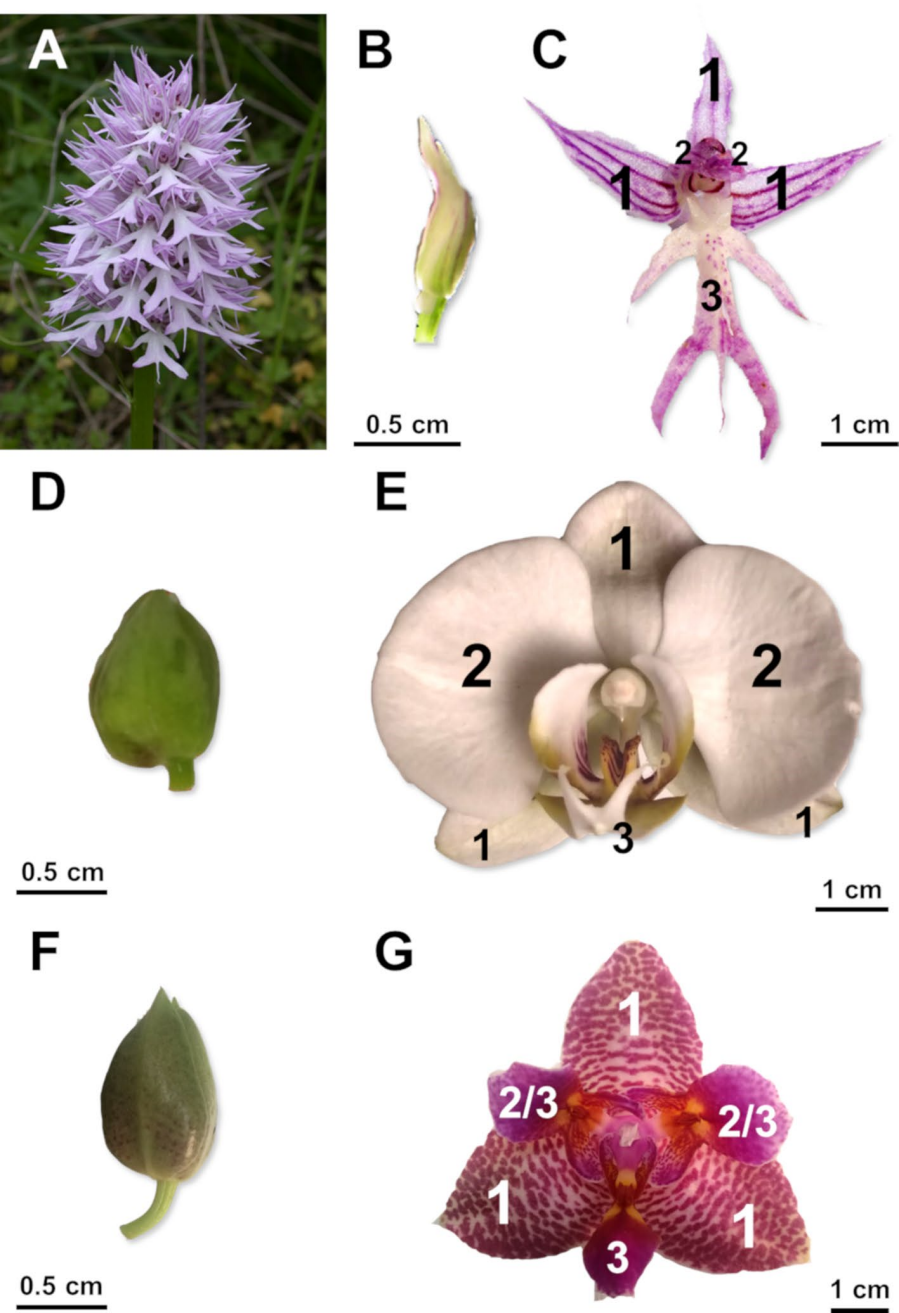
Flowers of zygomorphic and peloric orchids. *Orchis italica* (Orchidoideae): **(A)** inflorescence; single flower before **(B)** and after **(C)** anthesis. *Phalaenopsis equestris* (Epidendroideae): flower before **(D)** and after **(E)** anthesis. *Phalaenopsis* Joy Fairy Tale: flower before **(F)** and after **(G)** anthesis. *1*, outer tepal; *2*, lateral inner tepal; *3*, lip; *2*/*3*, lip-like structure of *Phalaenopsis* Joy Fairy Tale.

Among monocots, Orchidaceae is one of the most species-rich families, adapted to many different habitats (Cozzolino and Widmer, 2005; Aceto and Gaudio, 2011). Most of the orchid species have zygomorphic flowers sharing a common organization of the perianth into three outer and three inner tepals. Zygomorphy of the orchid flower is evident in the diversified and complex morphology of the median inner tepal (labellum or lip; **Figure 2**) (Rudall and Bateman, 2002). The vast majority of studies concerning orchid flower development focused on MADS-box transcription factors (Salemme et al., 2011; Aceto et al., 2014; De Paolo et al., 2014; Cai et al., 2015; Lin et al., 2016; Zhang et al., 2016; Zhang et al., 2017; Valoroso et al., 2019), with particular attention to the *DEFICIENS* (*DEF*) and *AGAMOUS-LIKE 6* (*AGL6*) genes. The pivotal role of these genes in the evolution and formation of the orchid perianth is well explained by the “orchid code” theory and its successive modifications (Mondragon-Palomino and Theissen, 2009; Mondragon-Palomino and Theissen, 2011; Pan et al., 2011; Hsu et al., 2015; Dirks-Mulder et al., 2017). On the contrary, the MYB transcription factors are largely understudied in orchids and their potential involvement in the establishment of orchid flower symmetry has been only recently proposed in *Orchis italica* and *Cattleya trianae* (Valoroso et al., 2017; Madrigal et al., 2019).

To date, eight *DIV*, four *RAD*, and two *DRIF* genes have been reported in *O. italica* and, among them, the corresponding homologs responsible for floral symmetry of *A. majus* have been identified (Valoroso et al., 2017). Phylogeny and genomic organization of the orchid *DIV* and *RAD* genes has also been studied (Valoroso et al., 2017; Madrigal et al., 2019), whereas a description of the *DRIF* gene family is still missing.

The aim of the present study was to expand knowledge on *DIV*, *RAD*, and *DRIF* genes of orchids and to obtain more evidence supporting their involvement in the establishment of flower zygomorphy. We firstly focused on the orchid *DRIF* genes, searching for homologs within the orchid genomes and reconstructing their phylogeny. Then, we scanned the putative promoter and intron of the *DIV* and *RAD* genes to identify known *cis*-regulatory elements conserved between orchids and snapdragon. Finally, we analyzed the interaction ability of the OitDIV, OitRAD, and OitDRIF1 proteins of *O. italica* and examined their transcript abundance in the perianth tissues of zygomorphic and peloric orchid flowers.

## Materials and Methods

### Plant Material

The orchids used in this study were grown under natural light and temperature in the greenhouse of the Department of Biology of the University of Naples Federico II (Napoli, Italy). *O. italica* Poir. plants are part of the Orchidaceae collection of the Department of Biology. *Phalaenopsis equestris* (Schauer) Rchb.f. and *Phalaenopsis* Joy Fairy Tale (*Phal*. Ho’s Princess Arai × *Phal.* Coral Isles) are commercially available orchids (Giulio Celandroni Orchidee, San Giuliano Terme, Pisa, Italy). *O. italica* and *P. equestris* display flower zygomorphy as the second floral whorl is clearly distinguished into two lateral inner tepals and one median inner tepal (lip) (**Figures 2A–E**). The peloric perianth of *Phalaenopsis* Joy Fairy Tale shows two lip-like structures in substitution of the lateral inner tepals, conferring radial symmetry to the flower (**Figures 2F, G**).

Single flowers from three different plants of each orchid were collected before (single floret length, ∼1 cm) and soon after anthesis (**Figure 2**). The perianth tissues (outer tepals, inner lateral tepals, and lip) were dissected and stored in RNA-later (Ambion) until RNA extraction.

### Sequence Retrieval and Phylogenetic Analysis

In order to identify *DRIF* transcripts expressed in flower tissues of *O. italica*, the amino acid sequences corresponding to the DUF3755 domain of the known *O. italica* OitDRIF1 and two proteins (GenBank accession numbers MK834277 and MK834278, respectively) (Valoroso et al., 2017) were used as queries to scan the inflorescence transcriptome of *O. italica* (De Paolo et al., 2014) through tBLASTn searches. Using the same approach, *DRIF* homologs were searched within the transcriptomes of other orchids present in the *Orchidstra* database (Chao et al., 2017) and within the genome of *Apostasia shenzenica*, *P. equestris*, and *Dendrobium catenatum* (Cai et al., 2015; Zhang et al., 2016; Zhang et al., 2017). Recently identified *DRIF* nucleotide and amino acid sequences (Raimundo et al., 2018) were downloaded from SustainPine (http://www.scbi.uma.es/sustainpinedb/home_page), Monocots PLAZA (https://bioinformatics.psb.ugent.be/plaza/versions/plaza_v4_monocots/), NCBI (https://www.ncbi.nlm.nih.gov/), and Araport (https://www.araport.org/). The species and the corresponding accession numbers of the DRIF sequences used in the present work are listed in **Table S1**.

The amino acid sequences of the DRIF homologs identified were aligned with MAFFT (Katoh and Standley, 2013) and the resulting alignment was manually adjusted. Poorly aligned positions were removed using GBLOCKS (Talavera and Castresana, 2007) and maximum likelihood (ML) phylogenetic tree was constructed with RAxML v 8.2.10 (Stamatakis, 2014) using the default settings, with 1,000 bootstrap replicates.

### Analysis of Conserved Transcription Factor Binding Sites

Approximately 3 kb noncoding sequences upstream of the translation start site of the *DIV* and *RAD* genes of the orchids *A. shenzenica*, *D. catenatum*, and *P. equestris* were downloaded from the corresponding genomes deposited at NCBI (**Table S1**). These putative promoter sequences were scanned for the presence of conserved transcription factor binding sites (TFBSs) using the *PlantPAN 3.0* database (Chow et al., 2019). The Multiple Promoter Analysis search mode was applied to identify known conserved plant TFBSs shared within each gene group of putative promoters. Specific nucleotide motifs known as TFBSs of CYC (5′-GGNCCC-3′) (Kosugi and Ohashi, 2002; Costa et al., 2005; Yang et al., 2012; Gao et al., 2015; Sengupta and Hileman, 2018) and DIV (5′-VGATAMSV-3′) (Raimundo et al., 2013; Sengupta and Hileman, 2018) of *A. majus* were searched within the orchid *RAD* and *DIV* putative promoters, respectively. In addition, the intron sequences of the *RAD* genes of *O. italica*, *P. equestris*, *D. catenatum* (Valoroso et al., 2017), and *A. shenzenica* were scanned for the presence of the CYC TFBS, as described above.

### Expression Analysis

Total RNA was extracted from the perianth tissues (outer tepals, inner tepals, and lip, before and after anthesis) of *O. italica*, *P. equestris*, and *Phalaenopsis* Joy Fairy Tale using Trizol (Ambion) followed by DNase treatment. After RNA extraction and quantification, 500 ng of total RNA from each tissue were reverse-transcribed using the Advantage RT-PCR kit (Clontech) and a mix of oligo dT and random hexamer primers.

In order to validate the nucleotide sequence of the four *OitDRIF* transcripts identified in the inflorescence transcriptome of *O. italica*, specific primer pairs were designed (**Table S2**) and used to amplify the cDNA of *O. italica* inflorescence. The amplification products obtained were cloned into pSC-A-amp/kan vector (Agilent), sequenced using the T3 and T7 primers, and analyzed using an ABI 310 Automated Sequencer (Applied Biosystems). Their sequence was compared with that of the transcripts identified in the transcriptome of *O. italica*.

Relative expression of the orchid *DIV*, *RAD*, and *DRIF1* genes was evaluated by real-time PCR experiments, using 18S as reference gene, as previously described (De Paolo et al., 2015; Valoroso et al., 2017). Primer pairs are listed in the **Table S2**. Reactions were conducted in biological triplicates and technical duplicates. Mean ± SEM was calculated for each duplicate and biological triplicate. Gene relative expression level in inner lateral tepals and lip was normalized relative to outer tepals. Two-tailed *t* test was conducted to assess the statistical significance of the relative expression differences between lateral inner tepals and lip of each species, before and after anthesis.

### Yeast Two-Hybrid Analysis

The coding sequences (CDSs) of the *OitDIV* (KY089088), *OitRAD* (KY089097), and *OitDRIF1* (MK834277) homologs of *O. italica* were PCR amplified using the primer pairs listed in **Table S2** and 500 ng of cDNA of *O. italica* inflorescence. To analyse protein–protein interactions between OitDIV, OitRAD, and OitDRIF1, the GAL4-based yeast two-hybrid (Y2H) system (Matchmaker two-hybrid system; Clontech) was used. The amplified CDSs of *OitDIV*, *OitRAD*, and *OitDRIF1* were cloned into bait (pGBT9) and prey (pGAD424) vectors (Clontech). All the prey and bait recombinant vector combinations were used to transform *Saccharomyces cerevisiae* strain AH109 (Gietz et al., 1995), conducting each experiment in triplicate. Plasmid presence after double yeast transformations was checked by growing cells in Synthetic Defined (SD) medium lacking tryptophan and leucine. Protein ability to interact with each other was evaluated in SD medium lacking tryptophan, leucine, and histidine. Possible transcriptional activation activity of OitDIV, OitRAD, and OitDRIF1 proteins fused to the binding domain of GAL4 (pGBT9 vector) was verified by monitoring growth of yeast transformed cells in SD medium without histidine, in the presence of 10 mM 3-aminotriazole. Empty vectors pGBT9 or pGAD424 were transformed in combination with the recombinant vectors as negative controls.

## Results and Discussion

### Identification and Phylogenetic Analysis of the Orchid *DRIF* Genes

To date, the *DRIF* genes of orchids have been identified only in *O. italica*, where the expression pattern of *OitDRIF1* and *OitDRIF2* was analysed in floral tissues (Valoroso et al., 2017). Evolutionary analysis has demonstrated the ancient origin of the *DRIF* genes: they have been found (together with the *DIV* genes) from green algae to angiosperms. In angiosperms, the *DRIF* homolog number in the examined species is generally five (Raimundo et al., 2018). These findings led us to search for other *DRIF* genes expressed in *O. italica*, to identify their homologs in other orchids and to verify the number of *DRIF* genes within the available genome of orchid species, currently restricted to the only subfamilies Epidendroideae and Apostasioideae.

Among plants, the DUF3755 domain is unique to DRIF proteins (Raimundo et al., 2018), and when we used it as query to identify other *DRIFs* expressed in the inflorescence transcriptome of *O. italica* we found two different transcripts, in addition to those previously identified, named *OitDRIF3* and *OitDRIF4* (accession numbers MK834279 and MK834280, respectively). All the four *OitDRIF* transcripts of *O. italica* encode for proteins containing the N-terminal MYB domain and the C-terminal DUF3755 domain. Within the genome of *P. equestris* and *D. catenatum* (both belonging to the subfamily Epidendroideae), we found four and three *DRIF* genes, respectively. In the genome of *A. shenzenica*, belonging to the basal subfamily Apostasioideae, we found three *DRIF* genes. All these orchid *DRIF* genes encode for proteins containing the MYB and the DUF3755 domain.

To cover all the five subfamilies of Orchidaceae, we scanned the transcriptomes of *Ophrys sphegodes*, belonging to the same subfamily of *O. italica* (Orchidoideae), *Cypripedium formosanum* (Cypripedioideae), and *Vanilla planifolia* (Vanilloideae) present in the database *Orchidstra* (Chao et al., 2017), a transcriptomics collection for Orchidaceae. In *C. formosanum* we found four *DRIF* transcripts, three in *O. sphegodes* and two in *V. planifolia*. Although some of them are not full-length transcripts, missing part of the N-terminus, all contain both the MYB and DUF3755 domains. The graphical view of the amino acid alignment of the orchid DRIF proteins is reported in **Figure S1**, where the consensus sequences of the MYB and DUF3755 domains are shown.

**Figure 3:**
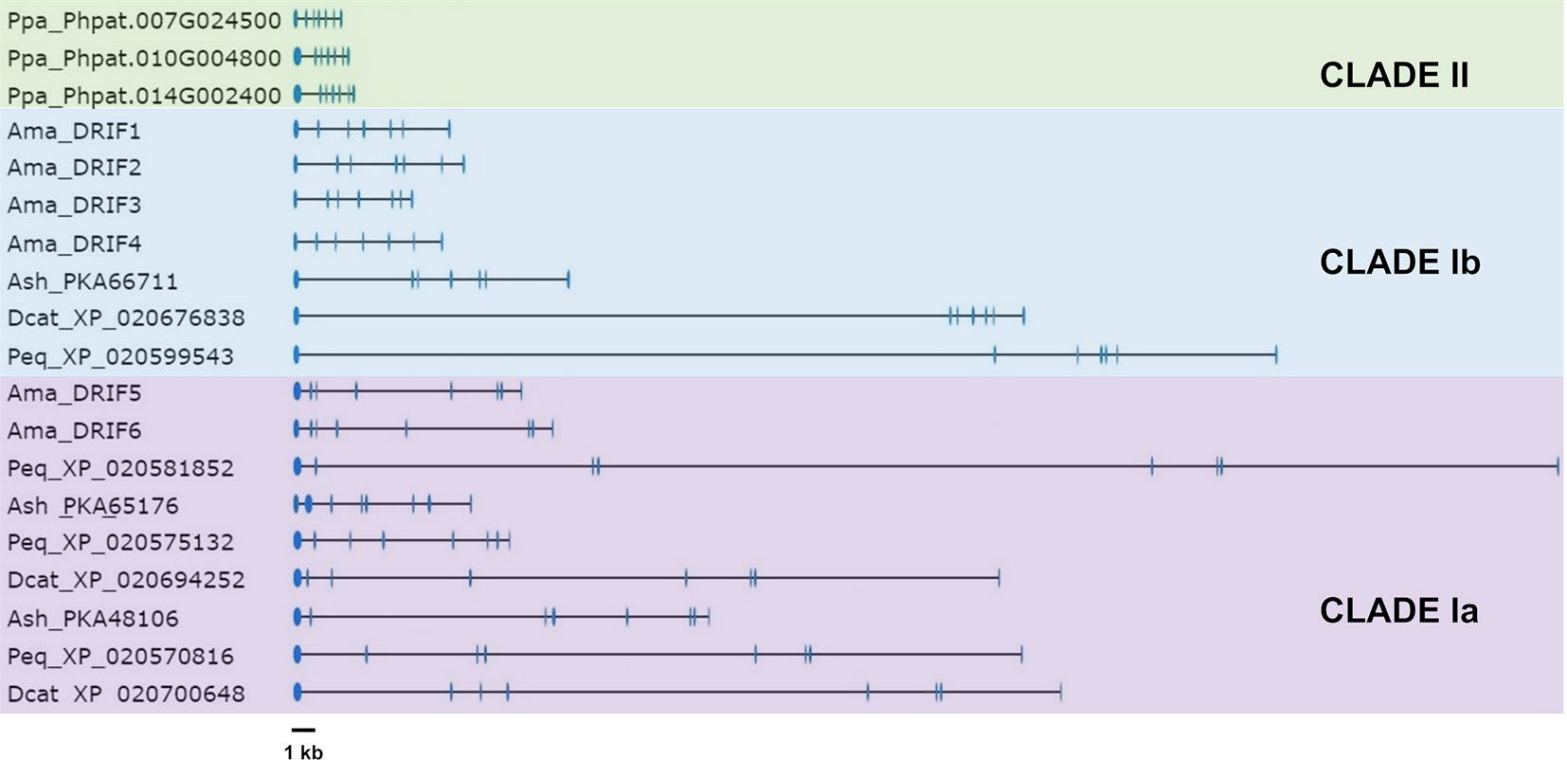
Genomic organization of the DRIF genes of *Phalaenopsis equestris*, *Dendrobium catenatum*, *Apostasia shenzenica* (Orchidaceae), *Antirrhinum majus* (Lamiales), and *Physcomitrella patens* (Bryophyta). *Light blue boxes* and *black lines* represent exons and introns, respectively. Ama, *A. majus*; Ash, *A. shenzenica*; Dcat, *D. catenatum*; Peq, *P. equestris*; Ppa, *P. patens*. Clades Ia, Ib, and II are referred to the main groups detected in the DRIF phylogeny (see figure). The code number following the abbreviation of the species name is the accession number of the DRIF sequences deposited in public databases (**Table S1**).

We reconstructed the genomic organization of the orchid *DRIF* genes from the assembled genomes of *P. equestris*, *D. catenatum*, and *A. shenzenica* and compared it to that of the *DRIF* genes of *A. majus*, whose genome assembly has been recently released (Li et al., 2019). Based on the exon/intron number, it is possible to divide the *DRIF* genes into two structural types: seven exons–six introns, shared by the *DRIF1-4* genes of *A. majus* and one *DRIF* gene of *A. shenzenica*, *D. catenatum*, and *P. equestris*; eight exons–seven introns, displayed by the *DRIF5-6* genes of *A. majus*, two *DRIF* genes of *A. shenzenica* and *D. catenatum*, and three of *P. equestris* (**Figure 3** and **Table S3**). Exon size is quite well conserved both among orchids and between orchids and snapdragon, whereas intron size is variable, with very large introns in orchids, reflecting a common feature of the orchid genomes due to the high number of transposable elements (Salemme et al., 2013a; Salemme et al., 2013b; Cai et al., 2015; Zhang et al., 2016; Zhang et al., 2017).

**Figure 4:**
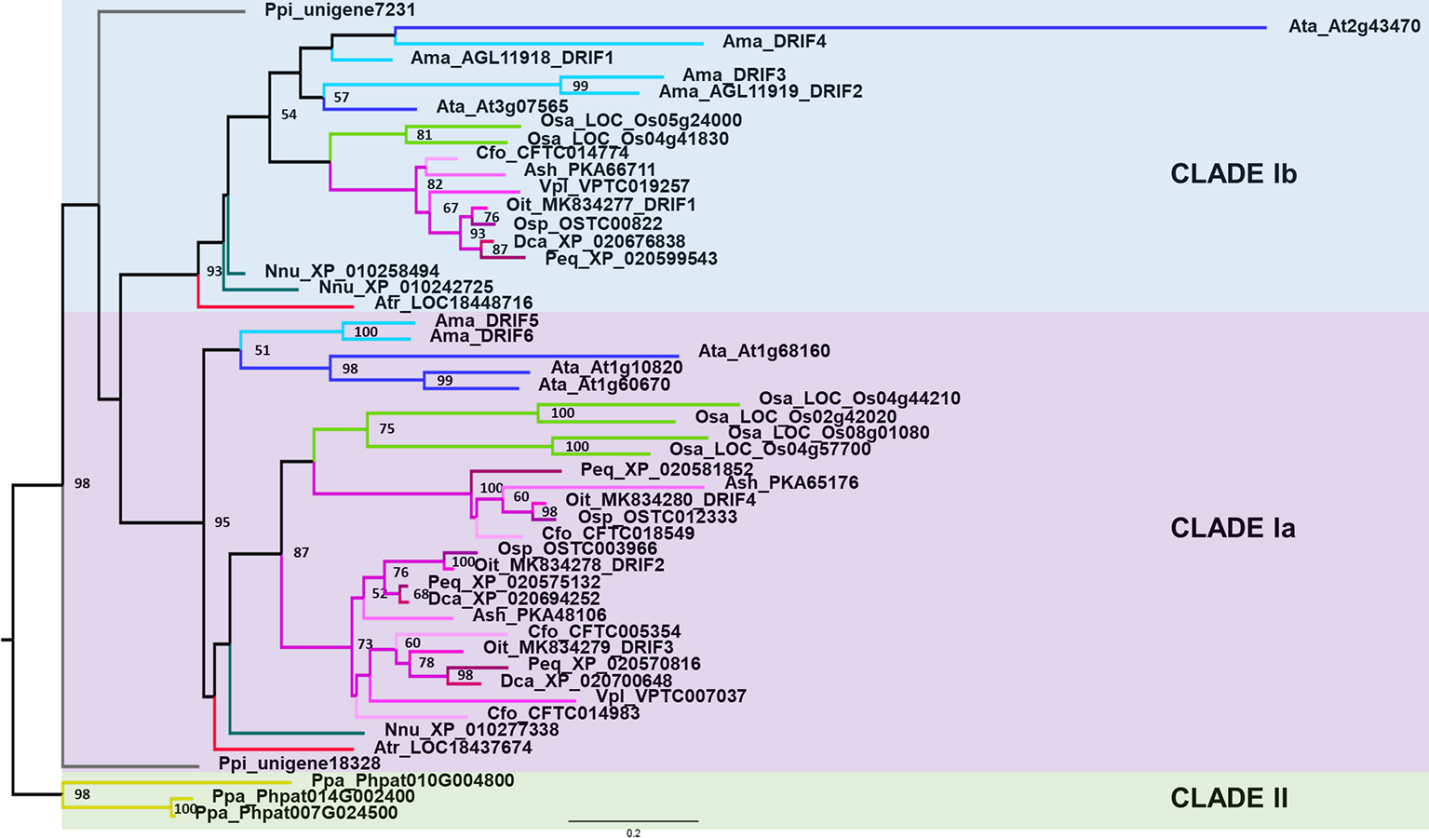
Maximum likelihood tree of the DRIF proteins of orchids and other plant species. The *numbers above the nodes* represent the bootstrap support percentages (1,000 replicates). Bootstrap values lower than 50% are not shown. The orchid branches are highlighted in *different shades of pink*. Ama, *Antirrhinum majus* (light blue); Ata, *Arabidopsis thaliana* (*blue*); Atr, *Amborella tricopoda* (*red*); Ash, *Apostasia shenzenica*; Cfo, *Cypripedium formosanum*; Dca, *Dendrobium catenatum*; Nnu, *Nelumbo nucifera* (*dark green*); Oit, *Orchis italica*; Osa, *Oryza sativa* (*green*); Osp, *Ophrys sphegodes*; Peq, *Phalaenopsis equestris*; Ppa, *Physcomitrella patens* (*yellow*); Ppi, *Pinus pinaster* (*gray*); Vpl, *Vanilla planifolia*. The code number following the abbreviation of the species name is the accession number of the DRIF sequences deposited in public databases (**Table S1**).

To understand the evolutionary relationships among the orchid DRIF proteins and the DRIFs of other plant species, we constructed the phylogenetic tree shown in **Figure 4**. The DRIF proteins of Tracheophyta are included in the clade I, whereas the three DRIF sequences of the moss *Physcomitrella patens* (Bryophyta) belong to the ancestral clade II, in agreement with the DRIF phylogeny recently described (Raimundo et al., 2018). Within the clade I, the orchid DRIFs (in the phylogenetic tree highlighted in different shades of pink) form three distinct groups, two belonging to the subclade Ia and one to the subclade Ib (**Figure 4**). Based on the tree topology, the orchid DRIFs seem to have originated by duplication events predating the diversification between monocots and dicots. In fact, within both subclades Ia and Ib, the orchid branches are grouped with other monocots (*O. sativa*, highlighted in green) and dicots (e.g., *A. majus* and *A. thaliana*, highlighted in different shades of blue). However, lineage-specific duplications have occurred during the evolution of DRIFs in orchids, leading to the formation of two paralog groups within the subclade Ia. In particular, an orchid-specific duplication generated the paralog groups that include, among others, OitDRIF2 and 3. The orchid homologs of DRIF1 of *A. majus* belong to the subclade Ib, where orchid-specific duplication events seem to be absent. In fact, within the subclade Ib, a single DRIF is present for each orchid species, including OitDRIF1 of *O. italica*, and the orchid DRIF phylogeny reflects the evolutionary relationships existing within Orchidaceae: Apostasioideae, Cypripedioideae, and Vanilloideae are the most basal subfamilies, Epidendroideae and Orchidoideae the most derivates (Givnish et al., 2015). The phylogenetic relationships among DRIF proteins coincide with the division proposed based on the genomic organization of the *DRIF* loci examined, showing that the genes belonging to the subclade Ia have eight exons, whereas those belonging to the subclade Ib have seven exons. The genomic organization of the *DRIF* genes of the moss *P. patens*, belonging to the basal clade II, shows the presence of seven exons and six introns (**Figure 3** and **Table S3**). The exon/intron structure shared by genes belonging to clade II and subclade Ib indicates that this genomic organization might be the ancestral condition and that the subclade Ia might have originated through a split of exon 7, followed by lineage-specific evolution of exon size.

Compared to the *DRIF* genomic organization, *DIV* and *RAD* genes have a significantly different structure with two exons and one intron (Valoroso et al., 2017). This difference supports the hypothesis previously proposed on the evolutionary origin of the *DIV*, *RAD*, and *DRIF* genes based on the comparison of their MYB domain (Raimundo et al., 2018). The *RAD* genes might have originated through the loss of the region of the *DIV* genes encoding for the MYBII domain. The *DIV* and *DRIF* genes might have evolved from a common ancestral gene through lineage-specific rearrangements (e.g., duplications or gene fusion events), resulting in different exon/intron organizations and acquisition of the region encoding for the MYBII (*DIV* lineage) or DUF3755 domain (*DRIF* lineage). Phylogenetic trees of the DIV and RAD proteins of orchids and other plant species are reported in **Figures S2** and **S3**, respectively. Both the DIV and RAD trees have been produced by the same approach used for the construction of the DRIF tree (see *Materials and Methods*).

### Analysis of the Conserved TFBS

Some aspects of transcriptional regulation of the genes involved in floral bilateral symmetry are known for *DIV* and *RAD* of *A. majus*. In snapdragon, the expression of the *RAD* gene is activated by direct interaction of the TCP transcription factor CYC possibly through the binding to three conserved TFBSs, two located within the promoter and one within the intron of *RAD* (Costa et al., 2005). The presence of conserved CYC TFBSs within the promoter of *RAD* has been recently reported also in other Lamiales species (Sengupta and Hileman, 2018).

Within the genomic sequence upstream of the translation start site of the *RAD* gene of *P. equestris*, *D. catenatum*, and *A. shenzenica*, we found distinct conserved TFBSs (**Table S4**), among which TCP binding sites. The sequence 5′-GGNCCN-3′, very similar to the *A. majus* CYC consensus binding site 5′-GGNCCC-3′, is present in the putative *RAD* promoter of *A. shenzenica* (three sites) and *D. catenatum* (two sites) (**Table 1**). Its absence in *P. equestris* is possibly due to the lack of a complete sequence information of the upstream region (only 958 bp are currently available) of the *RAD* gene in the corresponding genomic scaffold. Within the *RAD* intron, the sequence 5′-GGNCCN-3′ is present in *D. catenatum* (four sites), *P. equestris* (one site), and *O. italica* (two sites), whereas in *A. shenzenica* it is not present. In *D. catenatum* and *P. equestris* one of the sites exactly matches the canonical CYC TFBS of *A. majus*. The presence of putative CYC target sequences within the promoter and intron of the *RAD* gene (**Table 1**) may suggest a conserved direct transcriptional regulation of *RAD* by CYC in zygomprphic orchid flowers. In *P. equestris*, *D. catenatum*, and possibly *O. italica* both promoter and intron *cis*-regulatory motifs might be necessary to activate the transcription of *RAD* in the specific spatial domain linked to zygomorphy, as in *A. majus*. The absence of these regulatory sequences within the *RAD* intron of *A. shenzenica* is in agreement with this hypothesis, *A. shenzenica* being a basal orchid species with radially symmetric perianth (Zhang et al., 2017). The putative CYC binding sequence of orchids diverged to some extent from that of *A. majus* and other Lamiales and possibly the orchid CYC protein co-evolved to recognize slightly different sequences.

**Table 1:** Predicted binding sites of CYC (5′-GGNCCC-3′) and DIV (5′-VGATAMSV-3′) of *Antirrhinum majus* in the putative promoter and intron of *RAD* and in the putative promoter of *DIV*, respectively, in the orchids *Phalaenopsis equestris*, *Dendrobium catenatum*, and *Apostasia shenzenica.*

Gene	Species	Sequence	Position	Strand	Feature
***RAD***	*Dendrobium catenatum*	GGTCCA	−1331	+	Putative promoter
		GGTCCA	−2924	+	
	*Apostasia shenzhenica*	GGTCCT	−799	+	
		GGACCA	−1026	+	
		GGGCCG	−2376	+	
	*Phalaenopsis equestris*	**GGACCC**	1019	+	Intron
	*Dendrobium catenatum*	GGACCT	123	+	
		GGACCT	218	+	
		**GGTCCC**	789	+	
		GGACCG	809	+	
	*Orchis_italica*	GGCCCG	503	+	
		GGCCCG	527	+	
***DIV***	*Phalaenopsis equestris*	AGATAAAG	−573	−	Putative promoter
		AGATAATA	−1551	+	
		AGATAAAA	−1685	−	
	*Dendrobium catenatum*	**CGATAACC**	−2210	−	
	*Apostasia shenzhenica*	**AGATAAGA**	−739	+	
		**CGATAAGA**	−1008	+	
		**GGATAAGA**	−2898	+	

The analysis of the RAD intron was conducted also in Orchis italica. The nucleotide positions of the putative promoters are indicated with negative numbers, considering as +1 the first nucleotide of the translation start site codon ATG. The nucleotide positions of the orchid RAD introns are numbered considering as +1 the first nucleotide of the intron sequence. The binding sites exactly conserved among orchids and snapdragon are in bold.

In *A. majus*, two putative DIV binding sites have been identified *in silico* within the *DIV* promoter, suggesting the existence of an autoregulatory loop that maintains the transcription of *DIV* (Sengupta and Hileman, 2018). Within the genomic sequence upstream the translation start site of the *DIV* gene of *P. equestris*, *D. catenatum*, and *A. shenzenica* many conserved TFBSs are present, among which MYB binding sites (**Table S4**). The target binding sequence of DIV of *A. majus* 5′-VGATAMSV-3′ is present in *A. shenzenica* (three sites) and *D. catenatum* (one site), whereas the three sequences found in *P. equestris* have A or T instead of C or G in the seventh position (**Table 1**). Although the DIV binding site of orchids is only partially conserved, these results suggest that also in orchids the transcriptional activity of *DIV* might be regulated by a positive feedback.

### Protein Interactions and Expression Pattern

In flowering plants, the involvement of the DRIF/DIV and DRIF/RAD complexes in floral zygomorphy has been demonstrated in *A. majus* (Raimundo et al., 2013) and inferred in a few other species (Garces et al., 2016; Madrigal et al., 2019).

**Figure 5:**
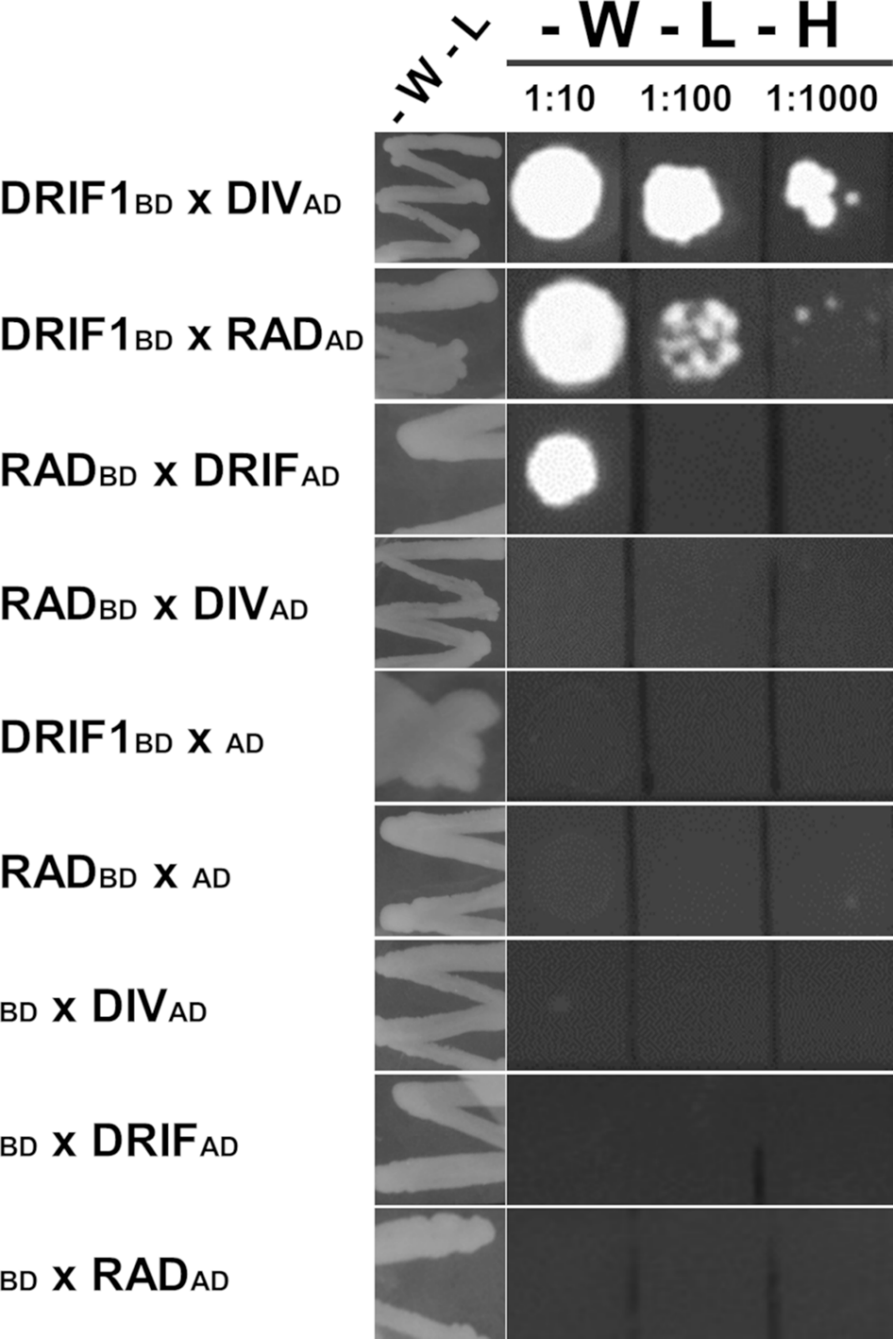
Interactions of the OitDIV, OitDRIF1, and OitRAD proteins of *Orchis italica* in Y2H analysis. After double transformations, yeast growth in absence of tryptophan and leucine (*-W-L*) indicates the plasmid presence; yeast growth in medium lacking tryptophan, leucine, and histidine denotes a positive interaction between the two tested proteins. Double transformations conducted using one of the vectors empty are negative controls. 1:10, 1:100, and 1:1,000 indicate the dilution factor applied to the yeast inoculate. *BD*, GAL4 DNA-binding domain (pGBT9 vector); *AD*, GAL4 activation domain (pGAD424 vector). As the OitDIV protein is able to promote transcription of the reporter genes (**Figure S4**), only its fusion to the GAL4 activation domain (pGAD424 vector) is reported in combination with OitRAD or OitDRIF fused to the GAL4 binding domain.

To date, the interaction among the DRIF, DIV, and RAD proteins in orchids has never been tested. We used the Y2H assay and found that in yeast the OitDRIF1 protein of *O. italica* can interact both with OitDIV and OitRAD, whereas OitDIV and OitRAD do not directly interact (**Figure 5**). This result is in agreement with the ancient evolutionary origin of this interaction module. In fact, the ability of the DIV and DRIF proteins to interact has evolved early, coincident with their origin in the green algae lineage. Later, with the emergence of the *RAD* genes in gymnosperms, the DRIF–RAD interaction has evolved (Raimundo et al., 2018).

**Figure 6:**
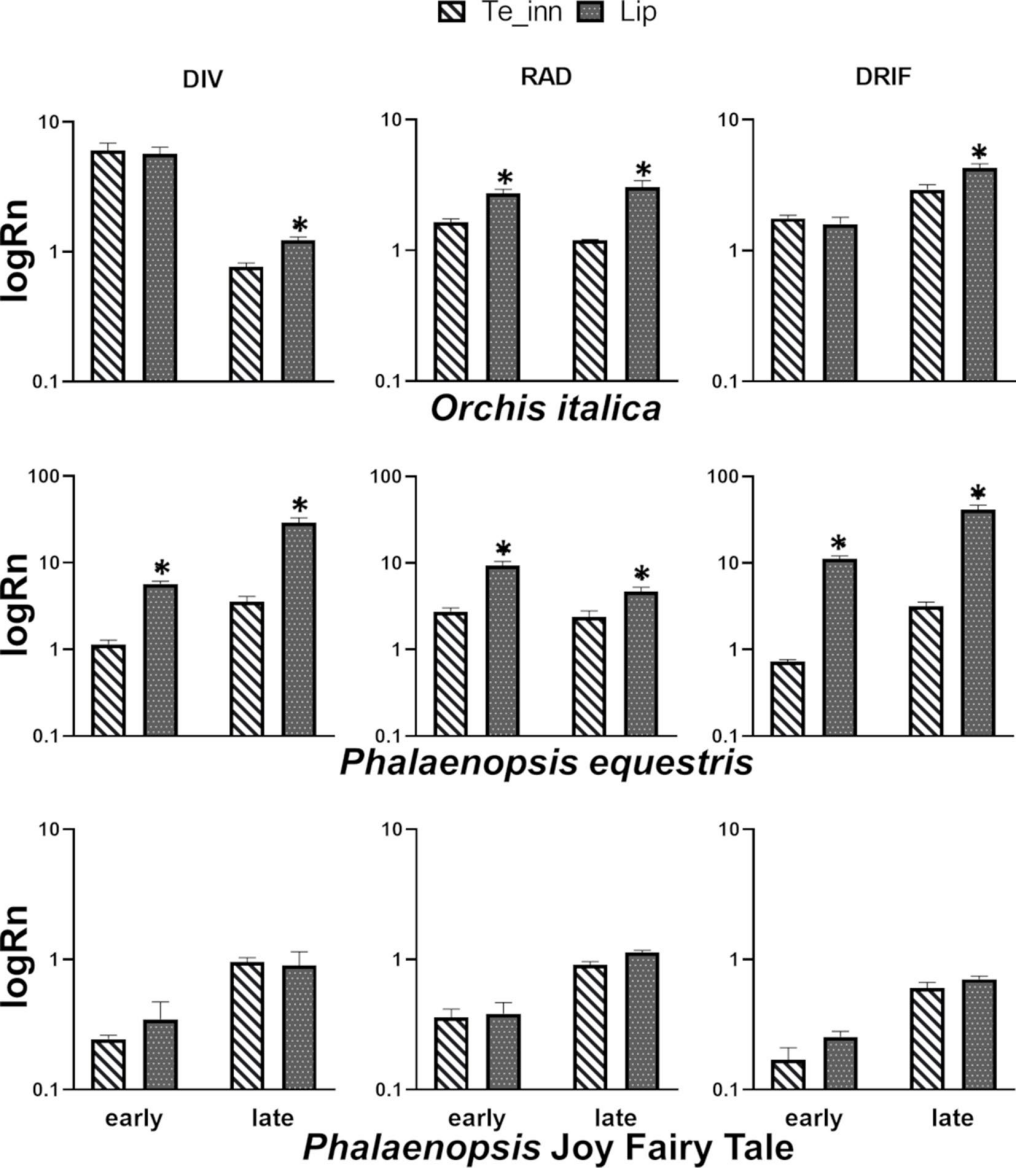
Relative expression of the orchid *DIV*, *RAD*, and *DRIF1* genes in lateral inner tepals (*Te_inn*) and lip of *Orchis italica*, *Phalaenopsis equestris*, and *Phalaenopsis* Joy Fairy Tale. The relative expression Rn (expressed as log_10_ of the mean between technical duplicates of three biological replicates) is normalized with respect to 18S endogenous gene and outer tepal tissue. The *bars* represent SEM and the *asterisks* indicate significant difference in relative expression between lateral inner tepals and lip assessed by *t* test. *Early* and *late* indicate before and after anthesis stage, respectively.

To obtain evidences about the possible involvement of the orchid *DIV*, *RAD*, and *DRIF* genes in zygomorphy of orchid flowers, we examined their expression profile in the perianth tissues of *O. italica* and *P. equestris*, both with zygomorphic flower, and in a peloric *Phalaenopsis* missing the bilateral symmetry due to the presence of three lips in the second floral whorl (**Figure 2G**). **Figure 6** shows the expression levels, before and after anthesis, of the orchid *DIV*, *RAD*, and *DRIF1* genes in inner tepals and lips normalized with respect to outer tepals. In the lip of *O. italica* and *P. equestris*, the expression level of the orchid *DIV*, *RAD*, and *DRIF1* is significantly higher than in inner tepals after anthesis. This same pattern is observed before anthesis for the three genes in *P. equestris* and only for *RAD* in *O. italica*, where *OitDIV* and *OitDRIF1* are expressed at similar levels in lip and inner tepals. This expression profile is in agreement with the previous reports about the expression of these genes in *O. italica* and *C. trianae* that suggested a possible involvement of the orchid *DIV*, *RAD*, and *DRIF1* in zygomorphy of the orchid perianth (Valoroso et al., 2017; Madrigal et al., 2019). Interestingly, the expression levels of *DIV*, *RAD*, and *DRIF1* in the peloric *Phalaenopsis* are similar in the lip and in the lip-like structures present in substitution of inner tepals, both before and after anthesis. The absence of a clear morphological differentiation among the structures of the second whorl in this peloric *Phalaenopsis* is associated with similar expression levels of the three genes, in particular of *RAD*. These results support the model in which in the zygomorphic flower of *O. italica* and *P. equestris*, DIV and DRIF, expressed in all the perianth organs as in *A. majus*, can interact in inner tepals, in which *RAD* expression is lower than in lip, and control ventralization. Regardless of DIV and DRIF1 levels, in the lip, RAD competes with DIV for the binding to DRIF1 and prevents the formation of the DIV–DRIF complex, thus inhibiting ventralization (Valoroso et al., 2017). Both in *O. italica* and *P. equestris*, the levels of *RAD* are lower in inner tepals than in lip. Consequently, in inner tepals, DIV could interact with DRIF1 and activate ventralization. On the contrary, the presence of higher levels of *RAD* in lip could allow the formation of the RAD/DRIF1 complex and inhibit the interaction between DIV and DRIF1, thus preventing ventralization. In the peloric *Phalaenopsis*, there is competition between DIV and RAD also in the lip-like structures of the second whorl and consequently ventralization is suppressed. The apparent rotation of the orchid model with respect to that of *A. majus* is due to resupination: the 180° rotation of the pedicel shifts the lip (a dorsal structure) to a ventral position during orchid flower development.

The results here obtained suggest the involvement of the orchid *DIV*, *RAD*, and *DRIF* genes in the zygomorphy of the orchid perianth and their conserved function, in addition to their conserved interaction ability, in species displaying flower zygomorphy.

Previous studies have demonstrated the role of the MADS-box genes in orchid flower development and various models have been proposed to explain the evolution and formation of the orchid perianth (Aceto and Gaudio, 2011; Salemme et al., 2013a; Acri-Nunes-Miranda and Mondragon-Palomino, 2014; Hsu et al., 2015; Dirks-Mulder et al., 2017; Valoroso et al., 2019). All these models propose interaction among different MADS-box transcription factors and attribute a crucial role to the expression levels of four *DEF* and three *AGL6* genes that permit or prevent the formation of specific protein quartets that drive the formation of specific parts of the perianth (outer tepals, inner tepals, lip) (Mondragon-Palomino and Theissen, 2009; Mondragon-Palomino and Theissen, 2011; Pan et al., 2011; Hsu et al., 2015; Dirks-Mulder et al., 2017). Our findings suggest the existence of a second, MYB-based pathway underlying flower organ arrangement in orchids.

The very recent advances in functional genetic studies of orchids (Kui et al., 2017) open the road to investigate the potential link between these two different molecular pathways (MADS- and MYB-based) in the formation of the orchid flower and its zygomorphy and to clarify if and what kind of interaction exists, to obtain an integrated view of this complex developmental process.

## Data Availability Statement

The datasets generated for this study can be found in the NCBI nucleotide, DRIF1: MK834277, DRIF2: MK834278, DRIF3: MK834279, DRIF4: MK834280.

## Author Contributions

MV performed the research, analyzed the data, and participated to write the paper. RS performed the research and analyzed the data. GS, MS, and MMRC analyzed the data and participated in the paper writing process. SA designed the research, analyzed the data, and wrote the paper.

## Funding

This study was financially supported by grant Ricerca dipartimentale 2018 from the University of Naples Federico II and by Fundação para a Ciência e Tecnologia/Ministério da Ciência, Tecnologia e Ensino Superior through national funds (Programa de Investimento e Despesas de Desenvolvimento da Administração Central) with a project grant PTDC/BIA-PLA/1402/2014 and by FCT/MCTES/PIDDAC (Portugal) under the project PEst-OE/BIA/UI4046/2014; UID/MULTI/04046/2013.

## Conflict of Interest

The authors declare that the research was conducted in the absence of any commercial or financial relationships that could be construed as a potential conflict of interest.
