# Supplementary figures and images for: Evolutionary Conservation of the Orchid MYB Transcription Factors DIV, RAD, and DRIF
Source: Front Plant Sci. 2019 Nov 1;10:1359. doi: 10.3389/fpls.2019.01359 (PMC6838138; doi:10.3389/fpls.2019.01359)

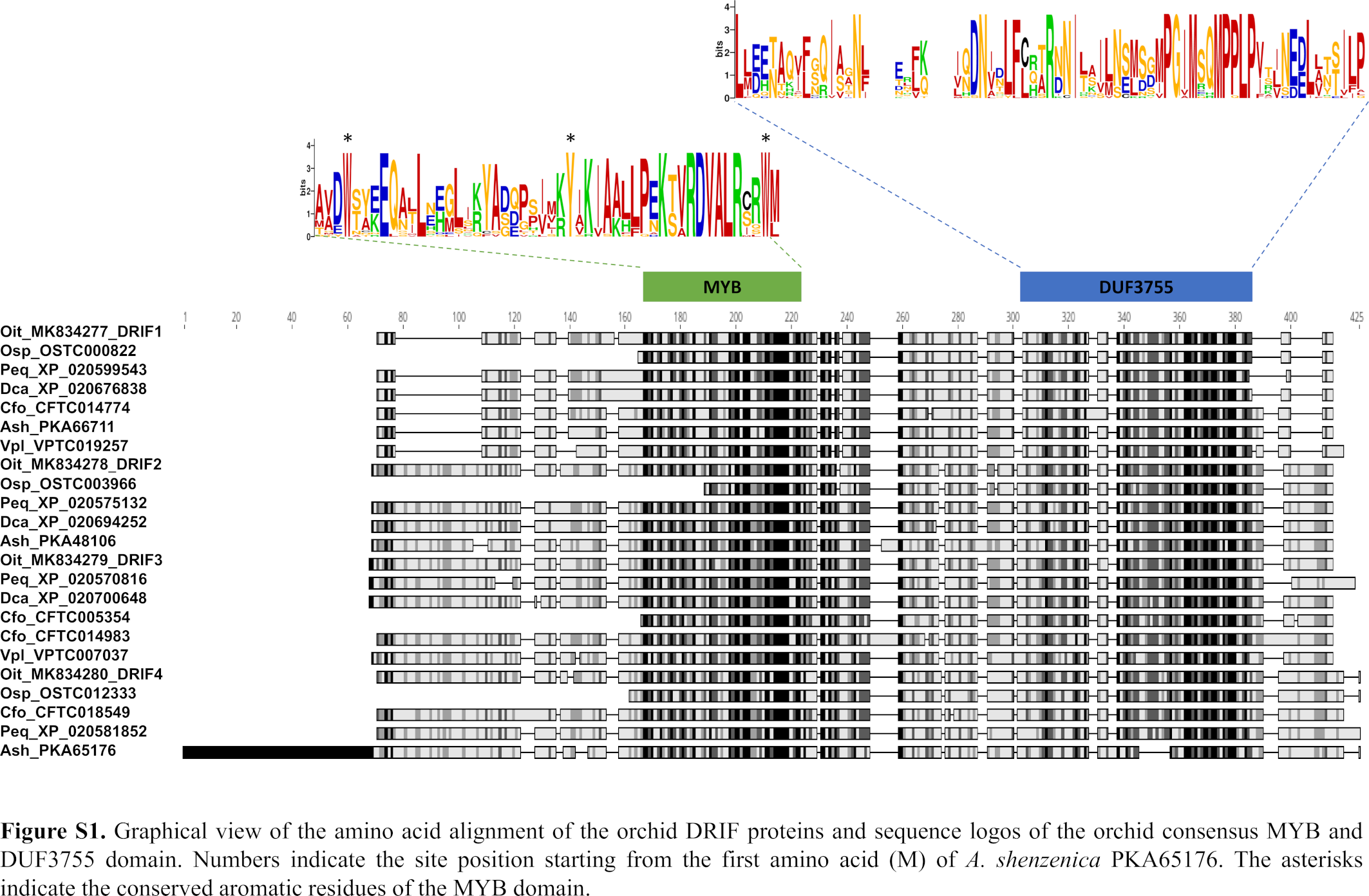

Supplement: Supplementary file 1 [file Image_1.tif]

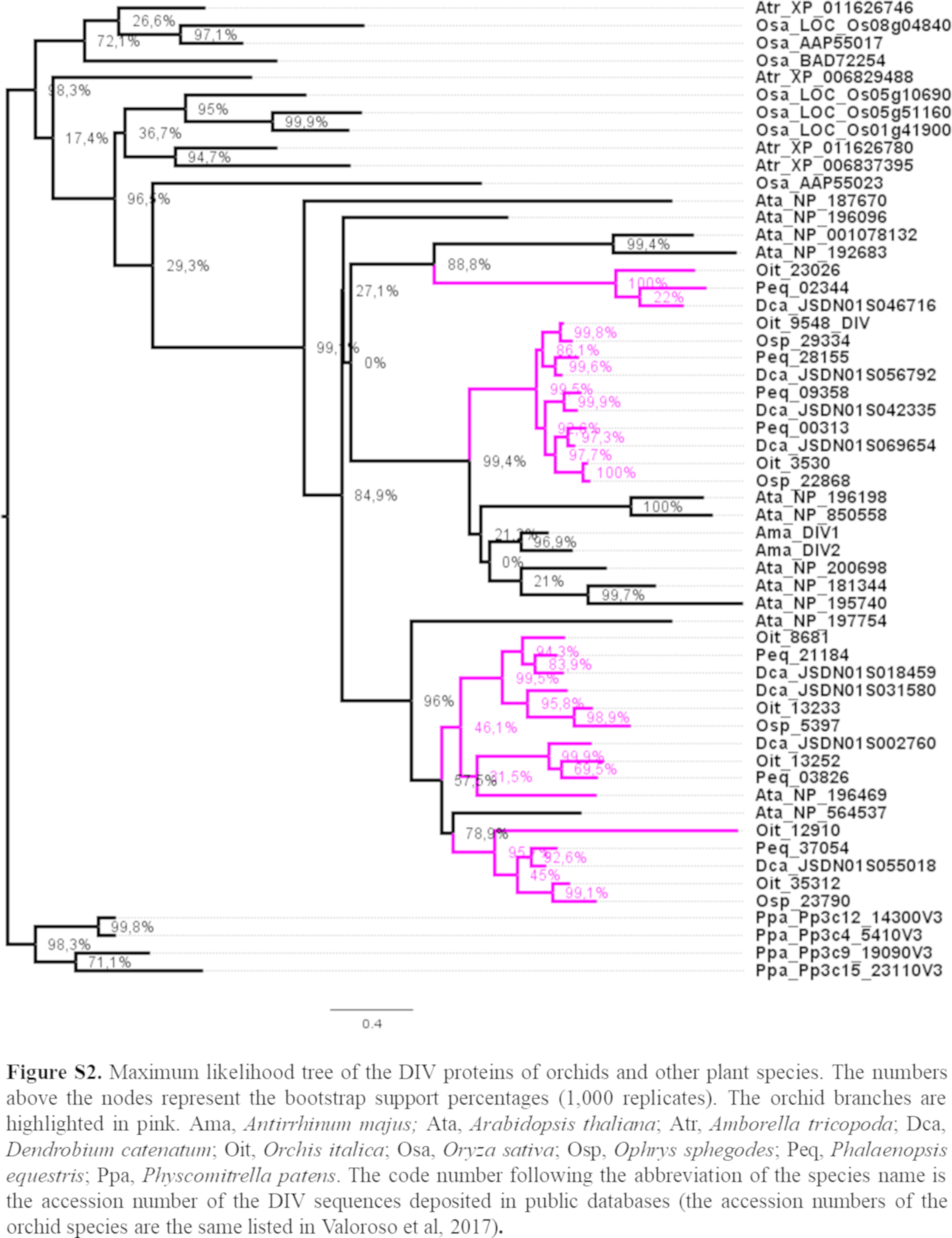

Supplement: Supplementary file 2 [file Image_2.tif]

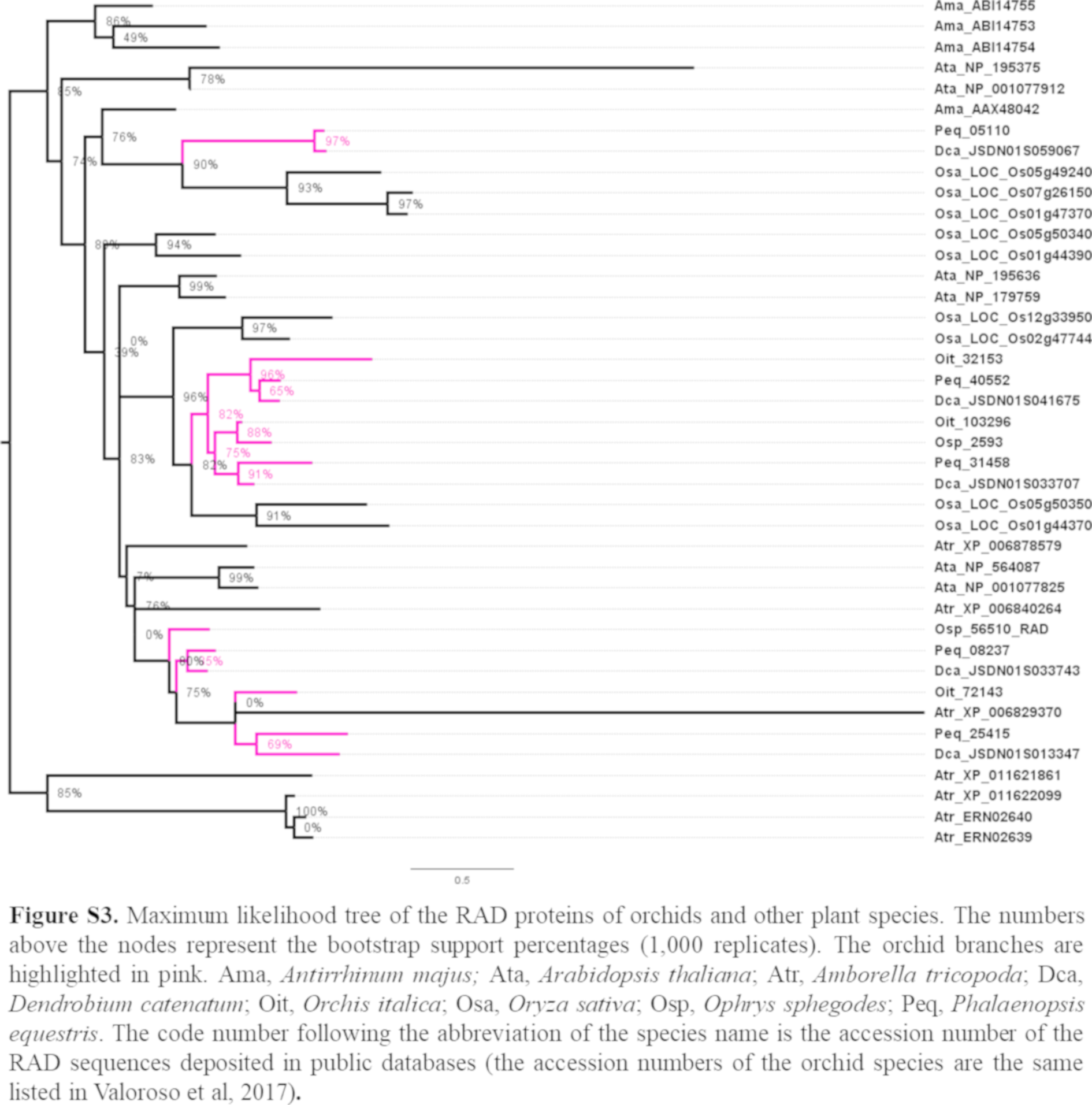

Supplement: Supplementary file 3 [file Image_3.tif]

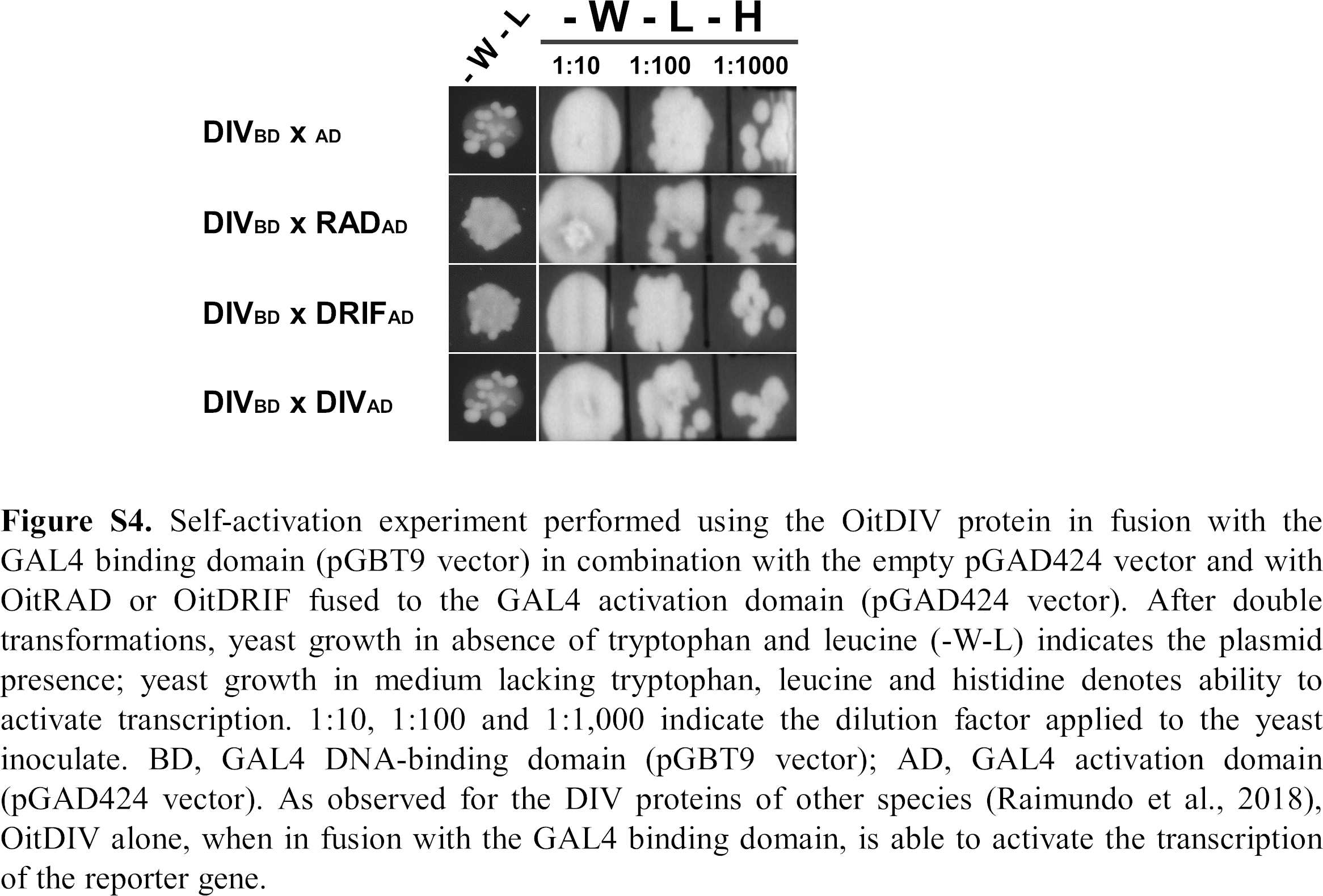

Supplement: Supplementary file 4 [file Image_4.tif]
